# Perceived traffic and road-surface exposures are associated with recent musculoskeletal complaints among motorcycle couriers

**DOI:** 10.1093/joccuh/uiag056

**Published:** 2026-09-18

**Authors:** Mehmet Akif Güler

**Affiliations:** Department of Physiotherapy and Rehabilitation, Faculty of Health Sciences, Selçuk University, Alaeddin Keykubat Campus, 299/1, 42250 Selçuklu, Konya, Türkiye

**Keywords:** motorcycle couriers, delivery riders, occupational health, ergonomics, musculoskeletal complaints, road-surface exposure

## Abstract

**Objectives:**

Motorcycle couriers work in traffic-intensive and physically demanding environments, but evidence on recent musculoskeletal complaints in this occupational group remains limited. This study estimated the 7-day prevalence of neck, shoulder, and low back complaints among motorcycle couriers and examined associations with self-reported working-condition indicators.

**Methods:**

A cross-sectional online survey was conducted among 286 actively working motorcycle couriers in Konya, Türkiye. Six perceived working-condition indicators—traffic, air pollution, noise, road irregularity/vibration, heat, and cold exposure—were assessed using 0-10 numeric rating scales. Recent neck, shoulder, and low back complaints were assessed separately for the previous 7 days. Associations were estimated using modified Poisson regression with robust variance. Perceived traffic exposure and perceived road irregularity/vibration exposure were prespecified as the main indicators of interest.

**Results:**

The 7-day prevalence of complaints was 55.9% for the neck, 52.8% for the shoulder, and 71.0% for the low back. In extended-adjusted models, higher perceived traffic exposure and higher perceived road irregularity/vibration exposure were consistently associated with all 3 complaint outcomes. These associations remained after false discovery rate correction, although they were attenuated in mutually adjusted sensitivity analyses. Secondary indicators showed more region-specific and less consistent associations.

**Conclusions:**

Recent musculoskeletal complaints were common among motorcycle couriers. Perceived traffic exposure and perceived road irregularity/vibration exposure were the most consistent self-reported working-condition indicators associated with reported complaints. These findings should not be interpreted as evidence that objectively measured occupational exposures contributed to the complaints and warrant confirmation in longitudinal studies using objective exposure assessment.

## Teaser

**Key points**
 

- **What is already known on this topic:** Motorcycle couriers and delivery riders are exposed to traffic density, prolonged riding, vibration, outdoor environmental conditions, and organizational work demands, but recent musculoskeletal complaints in this occupational group remain insufficiently studied.
- **What this study adds:** In a sample of 286 actively working motorcycle couriers, recent neck, shoulder, and low back complaints were common, and perceived traffic exposure and perceived road irregularity/vibration exposure showed the most consistent associations across anatomical regions.
- **How this study might affect research, practice, or policy:** The findings highlight perceived traffic- and road-surface-related working conditions as potential priorities for further occupational health research. Prospective studies using objective exposure measures are needed before specific preventive implications regarding traffic intensity, road-surface conditions, or riding-related vibration can be established.

## 1. Introduction

Online food delivery has become an increasingly visible component of urban transport systems and platform-mediated labor markets.^1^^,^^2^ In many cities, delivery riders spend long working hours in traffic, often under delivery-time pressure, income uncertainty, fatigue, and changing outdoor conditions.^2–4^ This work supports urban mobility and access to services, but it also places riders in an occupational setting where ergonomic load, environmental exposure, psychosocial demands, and road-related working conditions overlap.^2^^,^^5^ Motorcycle couriers and food delivery riders are therefore an important occupational group for occupational health and ergonomics research.

Studies from different countries have described long or irregular working hours, high delivery frequency, time pressure, fatigue, traffic hazards, crash risk, workload-related health problems, and limited occupational safety protection among riders working in platform-based and nonplatform delivery systems.^2^^,^^4^^,^^6–8^ Evidence from Türkiye is also emerging and points to occupational safety concerns, traffic-related injuries, protective equipment issues, psychosocial strain, and problematic working conditions among motorcycle couriers.^9–11^ These findings suggest that courier work should not be viewed only as an individual posture or fitness problem. It is also shaped by traffic density, road-surface quality, employment arrangements, delivery systems, climate, vehicle characteristics, and local occupational safety practices.

Musculoskeletal pain is a recognized health problem among professional drivers. A systematic review published in the *Journal of Occupational Health* reported that the prevalence of musculoskeletal pain among professional drivers ranged from 43.1% to 93%, with the low back being the most frequently affected region, followed by the neck and shoulder. The same review highlighted prolonged sitting, whole-body vibration, awkward posture, and repetitive actions as relevant occupational risk factors.^12^ An editorial in the same journal emphasized that musculoskeletal pain among professional drivers warrants prevention-oriented research and that more objective assessment of vibration, posture, sitting behavior, and driving duration is needed to clarify risk pathways and guide interventions.^13^

These mechanisms are plausible for motorcycle couriers. Riding requires prolonged sitting, trunk and neck stabilization, repeated head movements while monitoring traffic, continuous upper-limb control of the handlebars, and repeated loading and unloading during delivery tasks. These demands may combine with awkward posture, repetitive movements, long working hours, and vehicle-related factors that have been associated with work-related musculoskeletal problems among motorcycle food delivery riders.^14^^,^^15^ Poor road surfaces, speed bumps, potholes, and curb transitions may add further mechanical load through vibration and jolting. Objective measurement among taxi drivers has shown that whole-body vibration during actual working conditions may reach levels relevant to potential health risk, supporting the need to consider vibration and road-surface conditions in occupational driving research.^16^ More recent rider-specific evidence also suggests that daily whole-body vibration exposure among food delivery riders may exceed recommended exposure action values and may be related to work patterns and musculoskeletal disorders.^17^

Several gaps remain. Much of the available evidence on delivery riders has used 12-month symptom periods or broad definitions of work-related musculoskeletal disorders.^14^^,^^15^ Shorter recall periods may help describe more recent complaint burdens in relation to current working conditions. Evidence from Türkiye remains limited, and local courier work may differ from other settings because of traffic density, road-surface quality, platform organization, climate, employment arrangements, and occupational safety practices. In addition, few studies have examined traffic-related, environmental, and road-surface working conditions together in the same courier sample. This is important because motorcycle courier work is performed in a dynamic outdoor environment where traffic exposure, road irregularity, vibration, air pollution, noise, heat, and cold may coexist with ergonomic and organizational demands.

This study aimed to estimate the 7-day prevalence of neck, shoulder, and low back complaints among actively working motorcycle couriers in Konya, Türkiye, and to examine their associations with self-reported working-condition indicators. The research question was “Which indicators showed the most consistent associations with recent complaints across these 3 anatomical regions?” Self-reported traffic exposure and road irregularity/vibration exposure were considered the main indicators of interest because they are closely linked to the traffic and riding conditions of motorcycle courier work. Air pollution, noise, heat, and cold exposure were examined as secondary exploratory indicators. Because the study used a cross-sectional design and self-reported exposure and outcome measures, the findings were intended to identify preliminary occupational health signals and to inform future studies using longitudinal designs and objective exposure assessment.

## 2. Methods

### 2.1 Study design and setting

This cross-sectional survey was conducted among actively working motorcycle couriers in Konya, Türkiye. The study was reported in accordance with the Strengthening the Reporting of Observational Studies in Epidemiology statement.^18^ Ethical approval was obtained from the Non-Interventional Clinical Research Ethics Committee of Selçuk University Faculty of Health Sciences (Decision No. 2026/227). The study procedures were conducted in accordance with the principles of the Declaration of Helsinki. Electronic informed consent was obtained from all participants prior to data collection. Participation was voluntary and anonymous, and no names, phone numbers, email addresses, national identity numbers, license plate numbers, or other directly identifying information were collected. Formative interviews used to inform questionnaire development were conducted between March 1 and 5, 2026, and the main survey was conducted between March 6 and 31, 2026.

### 2.2 Participants and recruitment

Participants were eligible if they were aged 18 years or older, actively working as motorcycle couriers in Konya, and using a 2-wheeled motorized vehicle for delivery work. A nonprobability convenience sampling approach was used because no complete registry of motorcycle couriers was available. Potential participants were reached through courier peer networks. The online questionnaire was distributed via motorcycle courier WhatsApp groups and QR code invitations shared through field contacts. Eligibility was screened using self-reported age, current courier work status, vehicle type, and work location. Only submissions from participants who met the eligibility criteria were retained for analysis. The final analytic sample consisted of 286 actively working motorcycle couriers. All retained participants were male.

### 2.3 Questionnaire development and exposure indicators

The questionnaire was developed for this field-based survey to be brief, understandable, and feasible for actively working couriers. Before the main survey, semistructured formative interviews were conducted with 20 actively working motorcycle couriers in Konya to identify common traffic-related, road-surface, environmental, and workload-related conditions encountered during delivery work. These interviews were used to improve item relevance and wording; they were not designed as a formal qualitative study, and interviewees were not included in the analytic sample. Six study-specific working-condition indicators were assessed: traffic exposure, air pollution exposure, noise exposure, road irregularity/vibration exposure, heat exposure, and cold exposure. Each indicator was measured using a separate 0-10 numeric rating scale, where 0 indicated no or very low perceived exposure and 10 indicated very high perceived exposure. Road irregularity/vibration exposure referred to jolting or vibration perceived during riding because of uneven roads, speed bumps, potholes, and curb transitions. The indicators were analyzed separately because they were intended to represent distinct perceived aspects of the working environment rather than a unidimensional scale. The exact item wording, response options, and coding are provided in Data S1.

### 2.4 Outcome measures

The primary outcomes were self-reported neck, shoulder, and low back complaints during the previous 7 days. Each anatomical region was assessed separately using a binary yes/no item asking whether the participant had experienced pain, ache, or discomfort in that region during the preceding week. These outcomes represent recent self-reported complaints and not clinical diagnoses of musculoskeletal disorders.

Among participants reporting a complaint in a given region, worst pain intensity during the previous 7 days was assessed using an 11-point numeric rating scale ranging from 0, indicating no pain, to 10, indicating the worst pain imaginable. Numeric rating scales are commonly used for pain intensity assessment in clinical and research settings.^19^ Perceived impact on delivery work or ability to continue working was assessed for each symptomatic region using a 0-10 rating scale, where higher values indicated greater work impact. Assessment of pain-related interference is consistent with core outcome recommendations in pain research.^20^ Skip logic was used so that pain intensity and work-impact items were shown only for anatomical regions in which a participant reported a complaint, thereby reducing unnecessary response burden.^21^

### 2.5 Covariates

Covariates were selected a priori based on subject-matter relevance, plausibility as confounders, and availability in the questionnaire. They included age, courier experience, work type, average daily work duration, average daily riding duration, break frequency, average package weight, nightly sleep duration, self-rated stress during the previous 7 days, and achieving at least 150 minutes per week of moderate-intensity physical activity. Package weight was self-classified by participants as light, moderate, or heavy because objective package weights were not available. Because all participants were male, sex was described but not included in regression models. Education level and an alternative physical activity indicator, defined as at least 3 days per week and at least 30 minutes per day of moderate-intensity physical activity, were retained for descriptive and sensitivity analyses.

### 2.6 Data quality and missing data

Several procedures were used to improve data quality and reduce avoidable information bias. Participation was anonymous, response anchors were standardized across the working-condition indicators, skip logic was used to reduce unnecessary questionnaire burden, and the musculoskeletal complaint recall period was limited to 7 days. The Google Forms survey was configured to limit 1 response per account and required responses for the main eligibility, exposure, outcome, and covariate items. Therefore, analyses were conducted using complete eligible responses without imputation.

### 2.7 Study size

Sample size was calculated a priori for prevalence estimation using the single-proportion formula,^22^  *n* = Z^2^p(1 − p)/d^2^, where *n* is the required sample size, Z is the standard normal deviate corresponding to the desired confidence level, p is the expected prevalence, and d is the absolute precision. Because local prevalence data for motorcycle couriers were not available, the expected prevalence was based on a comparable study of motorcycle food delivery riders in Thailand that reported a low back pain prevalence of 33.3%.^15^ With a 95% CI and an absolute precision of 6%, the minimum required sample size was 237. The final analytic sample of 286 participants exceeded this minimum requirement. The study size calculation was designed for prevalence estimation; therefore, association analyses should be interpreted as exploratory.

### 2.8 Statistical analysis

Analyses were performed in R version 4.5.3. Continuous variables were summarized as mean ± SD or median with interquartile range, and categorical variables as number and percentage. The 7-day prevalence of neck, shoulder, and low back complaints was calculated separately. Perceived traffic exposure and perceived road irregularity/vibration exposure were prespecified as the main indicators of interest; the remaining perceived working-condition indicators—air pollution, noise, heat, and cold exposure—were analyzed as secondary exploratory indicators. Associations with each binary complaint outcome were estimated using modified Poisson regression with a log link and robust variance estimation.^23^ Results are reported as prevalence ratios with 95% CIs per 1-point increase on the 0-10 indicator scale.

For the main indicators, crude, minimal-adjusted, and extended-adjusted models were fitted. Minimal-adjusted models included age, courier experience, and work type. Extended-adjusted models additionally included daily work duration, daily riding duration, break frequency, package weight, sleep duration, self-rated stress score, and physical activity of at least 150 minutes per week. Spearman correlations were calculated among the 6 working-condition indicators, and multicollinearity was assessed using variance inflation factors and adjusted generalized variance inflation factors (Data S2, Tables S1 and S2).^24^ Benjamini-Hochberg false discovery rate correction was applied across the 18 extended-adjusted exposure-outcome associations.^25^ Sensitivity analyses included additional education adjustment, alternative coding of ordinal covariates, mutually adjusted exposure models, heteroscedasticity-consistent type 3 robust variance estimation, reduced-adjustment models excluding sleep duration, stress score, and physical activity, and tertile-based trend analyses. Interpretation emphasized consistency across anatomical regions, effect sizes, 95% CIs, and false discovery rate–adjusted results.

## 3. Results

### 3.1 Participant characteristics and 7-day complaint prevalence

The analytic sample included 286 actively working motorcycle couriers. All participants were male. Participant characteristics are summarized in Table 1. Recent musculoskeletal complaints were common, with low back complaints reported most frequently, followed by neck and shoulder complaints. The 7-day prevalence of complaints was 55.9% for the neck, 52.8% for the shoulder, and 71.0% for the low back (Table 2). Self-reported working-condition indicators showed moderate perceived exposure levels across traffic, air pollution, noise, road irregularity/vibration, heat, and cold conditions.

**Table 1:** Participant characteristics among motorcycle couriers.

Characteristic	Value
**Age, y**	27.97 ± 5.87
**Education level, *n* (%)**	
** Primary school**	32 (11.2)
** High school**	123 (43.0)
** Associate degree**	73 (25.5)
** Bachelor’s degree**	42 (14.7)
** Postgraduate**	16 (5.6)
**Courier experience, *n* (%)**	
** <6 months**	37 (12.9)
** 6-12 months**	70 (24.5)
** 1–3 years**	91 (31.8)
** >3 years**	88 (30.8)
**Work type, *n* (%)**	
** Platform-based**	154 (53.8)
** Company-based**	79 (27.6)
** Both**	53 (18.5)
**Daily work hours, *n* (%)**	
** <6 h**	43 (15.0)
** 6-8 h**	78 (27.3)
** 8-10 h**	122 (42.7)
** >10 h**	43 (15.0)
**Daily riding hours, *n* (%)**	
** <4 h**	34 (11.9)
** 4-6 h**	79 (27.6)
** 6-8 h**	108 (37.8)
** >8 h**	65 (22.7)
**Break frequency, *n* (%)**	
** Regular**	79 (27.6)
** Sometimes**	127 (44.4)
** Rarely**	80 (28.0)
**Package weight, *n* (%)**	
** Light**	44 (15.4)
** Moderate**	158 (55.2)
** Heavy**	84 (29.4)
** Sleep duration, h**	6.55 ± 0.95
** Stress score**	6.58 ± 2.13
** Physical activity ≥3 days/week, *n* (%)**	132 (46.2)
** Physical activity ≥150 min/week, *n* (%)**	77 (26.9)

Values are mean ± SD or *n* (%). All participants were male. Physical activity ≥3 days/week indicates ≥3 days/week and ≥30 min/day of moderate-intensity physical activity.

**Table 2:** Self-reported working-condition indicators and 7-day musculoskeletal complaint prevalence among motorcycle couriers.

Variable	Value
**Neck complaint, *n* (%)**	160 (55.9)
**Shoulder complaint, *n* (%)**	151 (52.8)
**Low back complaint, *n* (%)**	203 (71.0)
**Traffic exposure**	5.86 ± 3.29
**Air pollution exposure**	5.14 ± 2.92
**Noise exposure**	5.16 ± 3.00
**Road irregularity/vibration exposure**	5.48 ± 3.24
**Heat exposure**	4.39 ± 2.32
**Cold exposure**	5.30 ± 2.09

Values are mean ± SD or *n* (%). Exposure indicators were assessed on 0-10 numeric rating scales, with higher scores indicating higher perceived exposure.

### 3.2 Symptom intensity and work impact among symptomatic participants

Among participants reporting complaints, low back complaints showed the greatest symptom burden. The median worst pain intensity was 6 for low back complaints, compared with 5 for neck and shoulder complaints. Perceived work impact was also highest for low back complaints, followed by neck and shoulder complaints (Table 3).

**Table 3:** Symptom intensity and work impact among symptomatic motorcycle couriers.

Region	Participants with complaint, *n*	Worst pain intensity, median (IQR)	Worst pain intensity, mean ± SD	Work impact, median (IQR)	Work impact, mean ± SD
**Neck**	160	5 (4-6)	5.10 ± 1.84	4 (3–6)	4.39 ± 2.20
**Shoulder**	151	5 (3-6)	4.66 ± 2.21	4 (2–5.5)	3.99 ± 2.49
**Low back**	203	6 (4-7)	5.75 ± 2.11	5 (3-7)	4.78 ± 2.45

Abbreviation: IQR, interquartile range.

Only participants with the corresponding complaint were included.

### 3.3 Associations between working-condition indicators and 7-day complaints

The 2 prespecified perceived working-condition indicators, traffic exposure and road irregularity/vibration exposure, were consistently associated with all 3 complaint outcomes (Table 4 and Figure 1). These associations were observed in crude models and remained after minimal and extended adjustment. In extended-adjusted models, higher perceived traffic exposure was associated with higher prevalence of neck, shoulder, and low back complaints, with prevalence ratios ranging from 1.09 to 1.16 per 1-point increase on the 0-10 scale. Perceived road irregularity/vibration exposure showed a similar pattern, with extended-adjusted prevalence ratios ranging from 1.07 to 1.10 across the 3 anatomical regions.

**Table 4:** Primary associations between self-reported working-condition indicators and 7-day musculoskeletal complaints among motorcycle couriers.

Outcome	Exposure	Crude PR (95% CI)	Minimal-adjusted PR (95% CI)	Extended-adjusted PR (95% CI)
**Neck complaint**	Traffic exposure	1.12 (1.08-1.17)	1.13 (1.08-1.18)	1.09 (1.04-1.14)
**Neck complaint**	Road irregularity/vibration exposure	1.10 (1.06-1.14)	1.10 (1.06-1.14)	1.07 (1.03-1.11)
**Shoulder complaint**	Traffic exposure	1.16 (1.11-1.21)	1.15 (1.10-1.20)	1.12 (1.06-1.17)
**Shoulder complaint**	Road irregularity/vibration exposure	1.13 (1.08-1.17)	1.11 (1.07-1.16)	1.09 (1.05-1.14)
**Low back complaint**	Traffic exposure	1.17 (1.13-1.20)	1.16 (1.12-1.20)	1.16 (1.12-1.21)
**Low back complaint**	Road irregularity/vibration exposure	1.12 (1.09-1.15)	1.10 (1.07-1.14)	1.10 (1.07-1.14)

Abbreviation: PR, prevalence ratio.

Estimates are prevalence ratios with 95% CIs from modified Poisson regression models with robust variance. Each exposure was modeled separately, and estimates are expressed per 1-point increase on the 0-10 self-reported exposure scale. Minimal-adjusted models included age, courier experience, and work type. Extended-adjusted models additionally included daily work duration, daily riding duration, break frequency, package weight, sleep duration, self-rated stress score, and physical activity ≥150 min/week.

**Figure 1:**
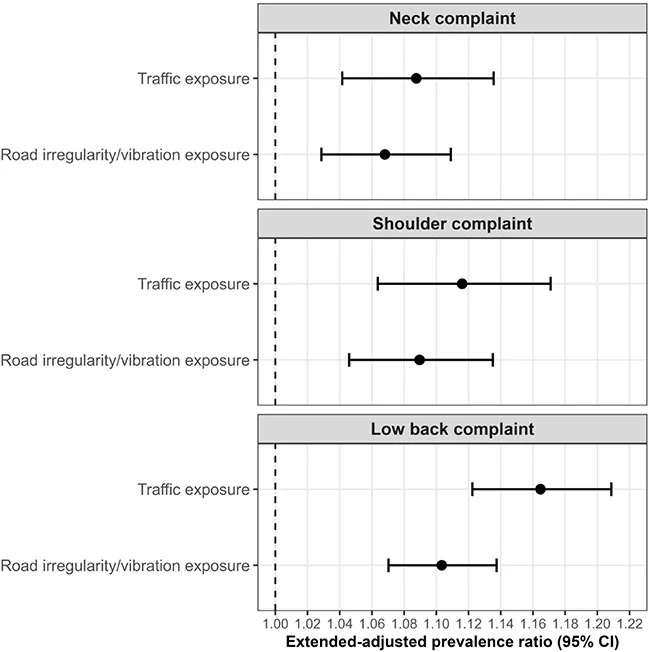
Extended-adjusted associations of the 2 primary self-reported working-condition indicators with 7-day musculoskeletal complaints among motorcycle couriers. Points represent prevalence ratios and horizontal lines represent 95% CIs. Estimates are from modified Poisson regression models with robust variance and are expressed per 1-point increase on the 0-10 exposure scale. Models were adjusted for demographic, work-related, sleep, stress, and physical activity covariates. The dashed vertical line indicates the null value.

Secondary exploratory associations varied by anatomical region (Table 5). In extended-adjusted models, perceived air pollution, noise, heat, and cold exposure indicators were associated with neck complaints. Perceived air pollution, noise, and heat exposure indicators were also associated with low back complaints. No secondary exploratory indicator showed clear evidence of association with shoulder complaints.

**Table 5:** Secondary exploratory associations between self-reported working-condition indicators and 7-day musculoskeletal complaints among motorcycle couriers.

Outcome	Exposure	Extended-adjusted PR (95% CI)	*P* value
**Neck complaint**	Air pollution exposure	1.08 (1.04-1.12)	<.001
**Neck complaint**	Noise exposure	1.06 (1.02-1.10)	.001
**Neck complaint**	Heat exposure	1.06 (1.01-1.10)	.016
**Neck complaint**	Cold exposure	1.08 (1.02-1.14)	.009
**Shoulder complaint**	Air pollution exposure	1.04 (1.00-1.07)	.070
**Shoulder complaint**	Noise exposure	1.03 (0.99-1.06)	.146
**Shoulder complaint**	Heat exposure	1.03 (0.99-1.07)	.189
**Shoulder complaint**	Cold exposure	1.02 (0.96-1.07)	.582
**Low back complaint**	Air pollution exposure	1.08 (1.05-1.11)	<.001
**Low back complaint**	Noise exposure	1.04 (1.01-1.07)	.003
**Low back complaint**	Heat exposure	1.05 (1.01-1.08)	.004
**Low back complaint**	Cold exposure	1.03 (1.00-1.07)	.078

Abbreviation: PR, prevalence ratio.

Estimates are prevalence ratios with 95% CIs from modified Poisson regression models with robust variance. Each exposure was modeled separately, and estimates are expressed per 1-point increase on the 0-10 self-reported exposure scale. Extended-adjusted models included age, courier experience, work type, daily work duration, daily riding duration, break frequency, package weight, sleep duration, self-rated stress score, and physical activity ≥150 min/week. These analyses were considered secondary and exploratory.

After Benjamini-Hochberg false discovery rate correction across the 18 extended-adjusted exposure-outcome tests, perceived traffic exposure and perceived road irregularity/vibration exposure remained associated with all 3 complaint outcomes. Among the secondary exploratory indicators, the associations retained after false discovery rate correction were those for neck complaints with air pollution, noise, heat, and cold exposure, and those for low back complaints with air pollution, noise, and heat exposure. None of the secondary exploratory indicators remained associated with shoulder complaints after false discovery rate correction (Data S2, Table S3).

### 3.4 Sensitivity analyses

Sensitivity analyses generally supported the robustness of the main findings. Additional adjustment for education level, alternative coding of ordinal covariates, heteroscedasticity-consistent type 3 robust variance estimation, and reduced-adjustment models excluding sleep duration, self-rated stress score, and physical activity produced broadly similar patterns. In exploratory mutually adjusted models including all 6 exposure indicators, associations were attenuated, particularly for neck complaints and for the secondary exploratory indicators. Perceived traffic exposure and perceived road irregularity/vibration exposure nevertheless remained the most consistent indicators, especially for shoulder and low back complaints (Data S2, Tables S4 and S5).

Tertile-based analyses broadly supported the main interpretation, although several indicators showed possible plateau or nonlinear patterns. Traffic exposure and road irregularity/vibration exposure showed the most consistent tertile-based patterns, particularly for shoulder and low back complaints, whereas the secondary exploratory indicators showed more region-specific or less consistent gradients (Data S2, Tables S6 and S7).

## 4. Discussion

### 4.1 Principal findings

This cross-sectional study examined recent neck, shoulder, and low back complaints among actively working motorcycle couriers and evaluated their associations with self-reported working-condition indicators. Recent musculoskeletal complaints were common, with the highest 7-day prevalence observed for low back complaints, followed by neck and shoulder complaints. The most consistent finding was that perceived traffic exposure and perceived road irregularity/vibration exposure were associated with all 3 complaint outcomes in extended-adjusted models. These associations remained after false discovery rate correction, although they were attenuated in mutually adjusted sensitivity analyses. Secondary exploratory indicators, including air pollution, noise, heat, and cold exposure, showed more region-specific and less consistent associations.

### 4.2 Comparison with previous studies

The high prevalence of recent complaints in this study is consistent with the broader occupational driving literature. A systematic review in professional drivers reported a high prevalence of musculoskeletal pain, with the low back, neck, and shoulder among the most commonly affected regions.^12^ The present findings are also consistent with evidence from gig-based food delivery workers and motorcycle food delivery riders, where musculoskeletal problems have been reported particularly in the low back, shoulder, and neck regions.^14^^,^^15^ These similarities suggest that musculoskeletal complaints among motorcycle couriers may share several mechanisms with other occupational driving groups, including prolonged sitting, repeated postural stabilization, vibration, awkward posture, repetitive upper-limb activity, and long working hours.^12^^,^^14^^,^^15^

At the same time, motorcycle courier work has specific features that may distinguish it from other forms of professional driving. Couriers work in dense and changing urban traffic, frequently stop and restart, monitor surrounding vehicles and pedestrians, stabilize the trunk and neck during riding, and control the motorcycle continuously with the upper limbs. Delivery work may also involve time pressure, fatigue, road-safety concerns, and variable outdoor environmental exposures. Previous studies among delivery riders have described job stress, fatigue, risky traffic conditions, crash-related concerns, and limited occupational safety protection.^2^^,^^4^^,^^6–8^ Evidence from Türkiye has also highlighted occupational safety concerns, traffic-related injuries, protective equipment issues, and problematic working conditions among motorcycle couriers.^9–11^ The present study adds to this literature by showing that recent musculoskeletal complaints are not only common but also associated with perceived traffic and road-surface working conditions.

The association between perceived road irregularity/vibration exposure and musculoskeletal complaints is occupationally plausible. Poor road surfaces, speed bumps, potholes, and curb transitions may increase repeated mechanical shock, whole-body vibration, trunk stabilization demands, and upper-limb effort during motorcycle control. Objective measurement studies among occupational drivers have shown that whole-body vibration during real working conditions may reach levels relevant to potential health risk.^16^ More recent rider-specific evidence also suggests that whole-body vibration exposure among food delivery riders may exceed recommended exposure action values and may be related to work patterns and musculoskeletal disorders.^17^ Although the present study did not objectively measure vibration or road-surface characteristics, the consistency of the perceived road irregularity/vibration indicator across anatomical regions suggests that objectively measured road-surface and vibration exposures warrant further investigation in motorcycle courier occupational health research.

Traffic exposure may also represent more than traffic density alone. In this study, perceived traffic exposure may have captured a demanding riding environment involving repeated braking and acceleration, sudden maneuvers, sustained vigilance, time pressure, perceived crash risk, and fatigue. These factors may increase physical and psychosocial load during delivery work. This interpretation is consistent with previous evidence showing that delivery riders experience traffic hazards, stress-related factors, fatigue, and road-safety challenges.^4^^,^^6^^,^^8^ However, because the exposure measure was self-reported, it should be interpreted as perceived traffic-related working demand rather than an objective measure of traffic density.

### 4.3 Occupational health and ergonomic implications

The findings suggest that motorcycle couriers’ perceptions of traffic- and road-surface-related working conditions may be relevant to their reported musculoskeletal complaints. However, because both the working-condition indicators and musculoskeletal outcomes were self-reported, the present study cannot determine whether objectively measured traffic intensity, road-surface quality, riding-related vibration, or other occupational exposures contributed to these complaints. Accordingly, the present findings should primarily be considered hypothesis-generating for occupational health research rather than evidence supporting specific preventive interventions. Future prospective studies incorporating objective measures of traffic conditions, road-surface characteristics, vibration, riding posture, and work organization are needed to determine whether these factors represent modifiable occupational risk factors.

The secondary exploratory findings should be interpreted cautiously. Air pollution, noise, heat, and cold exposure were more selectively associated with neck or low back complaints and did not show a consistent pattern across all anatomical regions. These indicators may reflect broader outdoor working conditions rather than direct musculoskeletal risk factors. Delivery riders work in environments where traffic emissions, noise, thermal conditions, and ergonomic demands coexist.^3^^,^^5^ However, because these indicators were self-reported and correlated with the main indicators, they should be viewed as hypothesis-generating. Future studies using objective environmental exposure measurement would help clarify whether these factors have independent or interactive roles in musculoskeletal complaint burden.

### 4.4 Strengths and limitations

This study has several strengths. It focused on an under-studied occupational group, used a recent 7-day recall period, assessed 3 anatomical regions separately, and distinguished between prespecified main indicators and secondary exploratory indicators. The use of modified Poisson regression, false discovery rate correction, collinearity diagnostics, and multiple sensitivity analyses strengthened the interpretation of the findings. The questionnaire was also informed by formative interviews with actively working couriers, which helped improve item relevance for the local field context.

Several limitations should also be considered. First, the cross-sectional design prevents causal inference. Couriers with musculoskeletal complaints may have perceived or reported their working conditions more negatively, and reverse reporting cannot be excluded. Second, recruitment through courier peer networks, WhatsApp groups, and QR code invitations may have introduced selection bias. Because no formal sampling frame was available, the number of eligible couriers who received or viewed the invitation was unknown and a response rate could not be calculated. Third, all retained participants were male and were recruited from a single city, which limits generalizability to female couriers, other regions, and other delivery systems. Fourth, the working-condition indicators were study-specific self-reported items and were not objective exposure measurements. Traffic density, road-surface quality, vibration magnitude, air pollution, noise, and thermal conditions were not measured directly. Therefore, exposure misclassification and common-method bias are possible. Accordingly, the observed associations should be interpreted as associations between participants’ perceptions of their working conditions and their reported musculoskeletal complaints, rather than as evidence of associations with objectively measured occupational exposures. Fifth, the outcomes were self-reported recent complaints rather than clinical diagnoses of musculoskeletal disorders. Although a short recall period may reduce recall burden, it does not establish chronicity or clinical severity. Sixth, package weight was self-classified rather than objectively measured, and information on previous musculoskeletal injury, secondary employment, motorcycle type, vehicle age, seat characteristics, suspension condition, protective equipment use, and detailed work organization was not available. Residual confounding is therefore possible. Finally, because data were collected in March, heat and cold indicators should be interpreted as perceived thermal conditions during the survey period rather than annual thermal exposure.

Future research should use longitudinal designs, representative or registry-supported sampling where feasible, and objective exposure assessment. Wearable sensors, vibration meters, route-based traffic indicators, environmental monitoring, and smartphone-based riding data could help quantify traffic exposure, riding duration, road-surface conditions, posture, and vibration more accurately. Such studies would help clarify causal pathways, identify modifiable risk factors, and develop targeted occupational health interventions for motorcycle couriers.

## 5. Conclusions

Recent neck, shoulder, and low back complaints were common among motorcycle couriers. Perceived traffic exposure and perceived road irregularity/vibration exposure were the most consistent self-reported working-condition indicators associated with reported complaints across all 3 anatomical regions. Perceived air pollution, noise, heat, and cold exposure indicators showed more region-specific exploratory associations. Given the cross-sectional design, self-reported exposure and outcome measures, and nonprobability sampling, these findings should be interpreted as associations between perceived working conditions and reported musculoskeletal complaints rather than as evidence that objectively measured occupational exposures contributed to these complaints. Future longitudinal studies incorporating objective measures of traffic conditions, road-surface characteristics, vibration, and riding-related ergonomic demands are needed to clarify these relationships and their potential preventive relevance.

## Supplementary Material

Supplementary_material_uiag056

## Data Availability

The data underlying this article cannot be shared publicly because participants did not provide consent for public data sharing and because of privacy considerations. Deidentified data may be made available from the corresponding author upon reasonable request and with appropriate ethics approval.
