# Supplementary material for: Perceived traffic and road-surface exposures are associated with recent musculoskeletal complaints among motorcycle couriers
Source: J Occup Health. 2026 Sep 18;68(1):uiag056. doi: 10.1093/joccuh/uiag056 (PMC13677067; doi:10.1093/joccuh/uiag056)
Supplement: Supplementary file 1 [file supplementary_material_uiag056.zip › Supplementary_Data_1_Questionnaire_Coding_Framework_uiag056.docx]

**Supplementary Data 1.** Study-specific questionnaire items and coding framework

**Article title:** Perceived Traffic and Road-Surface Exposures Are Associated With Recent Musculoskeletal Complaints Among Motorcycle Couriers

This supplementary file presents the study-specific working-condition indicators and musculoskeletal complaint items used in the survey. The working-condition indicators were developed for this field-based survey and were informed by formative interviews with actively working motorcycle couriers. Each working-condition indicator was assessed using a 0-10 numeric rating scale, where 0 indicated no or very low perceived exposure and 10 indicated very high perceived exposure. The indicators were analyzed separately and were not intended to form a single composite scale.

**Working-condition indicators**

| Indicator | English item wording | Response scale and coding |
| --- | --- | --- |
| Traffic exposure | During your work, how would you rate your level of exposure to traffic density? | 0-10 numeric rating scale; higher scores indicate higher perceived exposure |
| Air pollution exposure | During your work, how would you rate your level of exposure to exhaust fumes or polluted air? | 0-10 numeric rating scale; higher scores indicate higher perceived exposure |
| Noise exposure | During your work, how would you rate your level of exposure to noise? | 0-10 numeric rating scale; higher scores indicate higher perceived exposure |
| Road irregularity/vibration exposure | During your work, how would you rate your level of exposure to road irregularity/vibration, including jolting felt on the motorcycle because of uneven roads, speed bumps, potholes, and curb transitions? | 0-10 numeric rating scale; higher scores indicate higher perceived exposure |
| Heat exposure | During your work, how would you rate your level of exposure to heat or hot working conditions? | 0-10 numeric rating scale; higher scores indicate higher perceived exposure |
| Cold exposure | During your work, how would you rate your level of exposure to cold working conditions? | 0-10 numeric rating scale; higher scores indicate higher perceived exposure |

**Musculoskeletal complaint items**

Complaint items were assessed separately for the neck, shoulder, and low back regions. Each complaint item referred to the previous 7 days and was coded as 1 = yes and 0 = no.

| Region | English item wording | Response options and coding |
| --- | --- | --- |
| Neck complaint | During the past 7 days, have you experienced pain, ache, or discomfort in your neck? | Yes = 1; No = 0 |
| Shoulder complaint | During the past 7 days, have you experienced pain, ache, or discomfort in your shoulder region? | Yes = 1; No = 0 |
| Low back complaint | During the past 7 days, have you experienced pain, ache, or discomfort in your low back? | Yes = 1; No = 0 |

**Symptom intensity and work-impact items**

Among participants who reported a complaint in a given anatomical region, additional items assessed worst pain intensity and perceived work impact for that region. Skip logic was used so that these items were shown only for regions with a reported complaint.

| Item | English item wording | Response scale and coding |
| --- | --- | --- |
| Worst pain intensity | For each region with a reported complaint, how would you rate the worst pain intensity in that region during the past 7 days? | 0-10 numeric rating scale; 0 = no pain and 10 = worst pain imaginable |
| Work impact | For each region with a reported complaint, how much did this complaint affect your delivery work or ability to continue working during the past 7 days? | 0-10 numeric rating scale; 0 = no impact and 10 = very high impact |
