# Supplementary material for: Perceived traffic and road-surface exposures are associated with recent musculoskeletal complaints among motorcycle couriers
Source: J Occup Health. 2026 Sep 18;68(1):uiag056. doi: 10.1093/joccuh/uiag056 (PMC13677067; doi:10.1093/joccuh/uiag056)
Supplement: Supplementary file 1 [file supplementary_material_uiag056.zip › Supplementary_Data_2_Supplementary_Analyses_uiag056.docx]

**Supplementary Data 2.** Supplementary analyses

**Article title:** Perceived Traffic and Road-Surface Exposures Are Associated With Recent Musculoskeletal Complaints Among Motorcycle Couriers

This supplementary file presents correlation analyses, collinearity diagnostics, false discovery rate-adjusted results, and sensitivity analyses for the study.

**Supplementary Table S1.** Spearman correlations among self-reported working-condition indicators

| **Exposure indicator** | **Traffic exposure** | **Air pollution exposure** | **Noise exposure** | **Road irregularity/vibration exposure** | **Heat exposure** | **Cold exposure** |
| --- | --- | --- | --- | --- | --- | --- |
| Traffic exposure | 1.000 | 0.477 | 0.293 | 0.570 | 0.456 | 0.391 |
| Air pollution exposure | 0.477 | 1.000 | 0.665 | 0.320 | 0.197 | 0.324 |
| Noise exposure | 0.293 | 0.665 | 1.000 | 0.307 | 0.151 | 0.245 |
| Road irregularity/vibration exposure | 0.570 | 0.320 | 0.307 | 1.000 | 0.348 | 0.337 |
| Heat exposure | 0.456 | 0.197 | 0.151 | 0.348 | 1.000 | 0.374 |
| Cold exposure | 0.391 | 0.324 | 0.245 | 0.337 | 0.374 | 1.000 |

Values are Spearman’s rho. Higher scores indicate greater perceived exposure. All pairwise correlations between different exposure indicators were positive.

**Supplementary Table S2.** Collinearity diagnostics for the exploratory mutually adjusted model

| **Predictor** | **GVIF** | **Df** | **GVIF^(1/(2×Df))** |
| --- | --- | --- | --- |
| Traffic exposure | 1.819 | 1 | 1.349 |
| Air pollution exposure | 2.310 | 1 | 1.520 |
| Noise exposure | 1.965 | 1 | 1.402 |
| Road irregularity/vibration exposure | 1.535 | 1 | 1.239 |
| Heat exposure | 1.438 | 1 | 1.199 |
| Cold exposure | 1.545 | 1 | 1.243 |
| Age, years | 1.282 | 1 | 1.132 |
| Courier experience | 2.605 | 3 | 1.173 |
| Work type | 2.070 | 2 | 1.199 |
| Daily work hours | 2.787 | 3 | 1.186 |
| Daily riding hours | 2.836 | 3 | 1.190 |
| Break frequency | 2.319 | 2 | 1.234 |
| Package weight | 3.025 | 2 | 1.319 |
| Sleep duration | 1.435 | 1 | 1.198 |
| Stress score | 2.087 | 1 | 1.445 |
| Physical activity ≥150 min/week | 1.380 | 1 | 1.175 |

Variance inflation factors were calculated from an exploratory model including all six self-reported working-condition indicators and the prespecified covariates. For predictors with more than one degree of freedom, generalized variance inflation factors are shown together with adjusted GVIF values. The highest adjusted GVIF was 1.520 for air pollution exposure, indicating no evidence of problematic multicollinearity. df, degrees of freedom; GVIF, generalized variance inflation factor.

**Supplementary Table S3.** False discovery rate-adjusted *p* values for extended-adjusted associations

| Outcome | Exposure | Extended-adjusted *PR* (95% *CI*) | *p* value | FDR-adjusted *p* value |
| --- | --- | --- | --- | --- |
| Neck complaint | Traffic exposure | 1.09 (1.04–1.14) | <0.001 | <0.001 |
| Neck complaint | Road irregularity/vibration exposure | 1.07 (1.03–1.11) | <0.001 | 0.001 |
| Neck complaint | Air pollution exposure | 1.08 (1.04–1.12) | <0.001 | <0.001 |
| Neck complaint | Noise exposure | 1.06 (1.02–1.10) | 0.001 | 0.003 |
| Neck complaint | Heat exposure | 1.06 (1.01–1.10) | 0.016 | 0.022 |
| Neck complaint | Cold exposure | 1.08 (1.02–1.14) | 0.009 | 0.014 |
| Shoulder complaint | Traffic exposure | 1.12 (1.06–1.17) | <0.001 | <0.001 |
| Shoulder complaint | Road irregularity/vibration exposure | 1.09 (1.05–1.14) | <0.001 | <0.001 |
| Shoulder complaint | Air pollution exposure | 1.04 (1.00–1.07) | 0.070 | 0.090 |
| Shoulder complaint | Noise exposure | 1.03 (0.99–1.06) | 0.146 | 0.165 |
| Shoulder complaint | Heat exposure | 1.03 (0.99–1.07) | 0.189 | 0.200 |
| Shoulder complaint | Cold exposure | 1.02 (0.96–1.07) | 0.582 | 0.582 |
| Low back complaint | Traffic exposure | 1.16 (1.12–1.21) | <0.001 | <0.001 |
| Low back complaint | Road irregularity/vibration exposure | 1.10 (1.07–1.14) | <0.001 | <0.001 |
| Low back complaint | Air pollution exposure | 1.08 (1.05–1.11) | <0.001 | <0.001 |
| Low back complaint | Noise exposure | 1.04 (1.01–1.07) | 0.003 | 0.006 |
| Low back complaint | Heat exposure | 1.05 (1.01–1.08) | 0.004 | 0.006 |
| Low back complaint | Cold exposure | 1.03 (1.00–1.07) | 0.078 | 0.094 |

Estimates are prevalence ratios (PRs) with 95% confidence intervals (CIs) from extended-adjusted modified Poisson regression models with robust variance. Each exposure was modeled separately, and estimates are expressed per 1-point increase on the 0–10 self-reported exposure scale. Extended-adjusted models included age, courier experience, work type, daily work duration, daily riding duration, break frequency, package weight, sleep duration, self-rated stress score, and physical activity at least 150 minutes per week. False discovery rate-adjusted p values were calculated using the Benjamini–Hochberg procedure across the 18 exposure–outcome tests.

**Supplementary Table S4.** Comparison of primary and sensitivity analyses for adjusted associations between working-condition indicators and 7-day complaints

| Outcome | Exposure | Primary adjusted PR (95% CI) | Education-adjusted PR (95% CI) | Score-adjusted PR (95% CI) | Mutually adjusted PR (95% CI) | HC3-adjusted PR (95% CI) |
| --- | --- | --- | --- | --- | --- | --- |
| Neck complaint | Traffic exposure | 1.09 (1.04–1.14) | 1.09 (1.05–1.14) | 1.09 (1.04–1.14) | 1.05 (0.99–1.11) | 1.09 (1.04–1.14) |
| Neck complaint | Air pollution exposure | 1.08 (1.04–1.12) | 1.08 (1.04–1.12) | 1.08 (1.04–1.12) | 1.03 (0.97–1.09) | 1.08 (1.03–1.12) |
| Neck complaint | Noise exposure | 1.06 (1.02–1.10) | 1.06 (1.03–1.10) | 1.06 (1.02–1.09) | 1.03 (0.97–1.09) | 1.06 (1.02–1.10) |
| Neck complaint | Road irregularity/vibration exposure | 1.07 (1.03–1.11) | 1.07 (1.03–1.11) | 1.07 (1.03–1.11) | 1.03 (0.98–1.08) | 1.07 (1.03–1.11) |
| Neck complaint | Heat exposure | 1.06 (1.01–1.10) | 1.06 (1.01–1.11) | 1.05 (1.01–1.10) | 1.01 (0.96–1.05) | 1.06 (1.01–1.11) |
| Neck complaint | Cold exposure | 1.08 (1.02–1.14) | 1.09 (1.03–1.15) | 1.07 (1.01–1.12) | 1.04 (0.98–1.10) | 1.08 (1.01–1.15) |
| Shoulder complaint | Traffic exposure | 1.12 (1.06–1.17) | 1.11 (1.06–1.17) | 1.12 (1.07–1.18) | 1.10 (1.04–1.16) | 1.12 (1.06–1.18) |
| Shoulder complaint | Air pollution exposure | 1.04 (1.00–1.07) | 1.03 (1.00–1.07) | 1.04 (1.01–1.09) | 0.99 (0.93–1.04) | 1.04 (0.99–1.08) |
| Shoulder complaint | Noise exposure | 1.03 (0.99–1.06) | 1.03 (0.99–1.06) | 1.02 (0.98–1.06) | 1.02 (0.97–1.07) | 1.03 (0.99–1.07) |
| Shoulder complaint | Road irregularity/vibration exposure | 1.09 (1.05–1.14) | 1.09 (1.05–1.14) | 1.09 (1.04–1.13) | 1.05 (1.01–1.10) | 1.09 (1.04–1.14) |
| Shoulder complaint | Heat exposure | 1.03 (0.99–1.07) | 1.03 (0.99–1.07) | 1.03 (0.98–1.07) | 0.98 (0.94–1.02) | 1.03 (0.98–1.08) |
| Shoulder complaint | Cold exposure | 1.02 (0.96–1.07) | 1.02 (0.96–1.07) | 1.00 (0.96–1.06) | 0.98 (0.93–1.04) | 1.02 (0.96–1.08) |
| Low back complaint | Traffic exposure | 1.16 (1.12–1.21) | 1.16 (1.12–1.21) | 1.16 (1.12–1.21) | 1.14 (1.10–1.19) | 1.16 (1.12–1.21) |
| Low back complaint | Air pollution exposure | 1.08 (1.05–1.11) | 1.08 (1.04–1.11) | 1.08 (1.04–1.11) | 1.03 (0.99–1.06) | 1.08 (1.04–1.11) |
| Low back complaint | Noise exposure | 1.04 (1.01–1.07) | 1.04 (1.01–1.07) | 1.04 (1.01–1.07) | 1.00 (0.97–1.03) | 1.04 (1.01–1.08) |
| Low back complaint | Road irregularity/vibration exposure | 1.10 (1.07–1.14) | 1.10 (1.07–1.14) | 1.10 (1.07–1.13) | 1.05 (1.02–1.09) | 1.10 (1.07–1.14) |
| Low back complaint | Heat exposure | 1.05 (1.01–1.08) | 1.04 (1.01–1.07) | 1.05 (1.02–1.08) | 0.98 (0.95–1.00) | 1.05 (1.01–1.08) |
| Low back complaint | Cold exposure | 1.03 (1.00–1.07) | 1.03 (1.00–1.07) | 1.03 (1.00–1.07) | 0.98 (0.95–1.01) | 1.03 (0.99–1.07) |

Primary adjusted models included age, courier experience, work type, daily work duration, daily riding duration, break frequency, package weight, sleep duration, self-rated stress score, and physical activity ≥150 min/week. Education-adjusted models additionally included education level. Score-adjusted models entered ordinal covariates as ordinal scores rather than categorical variables. Mutually adjusted models included all six self-reported working-condition indicators simultaneously in addition to the primary covariates. HC3-adjusted models repeated the primary adjusted analyses using the HC3 robust variance estimator. Estimates are presented as prevalence ratios with 95% confidence intervals and are expressed per 1-point increase on the 0-10 self-reported exposure scale. CI, confidence interval; PR, prevalence ratio.

**Supplementary Table S5**. Reduced-adjustment sensitivity analyses for associations between working-condition indicators and 7-day complaints

| **Outcome** | **Exposure** | **Reduced-adjusted PR (95% CI)** | ***p*** |
| --- | --- | --- | --- |
| Neck complaint | Traffic exposure | 1.09 (1.04–1.13) | <0.001 |
| Neck complaint | Air pollution exposure | 1.08 (1.04–1.12) | <0.001 |
| Neck complaint | Noise exposure | 1.06 (1.03–1.10) | <0.001 |
| Neck complaint | Road irregularity/vibration exposure | 1.07 (1.03–1.11) | <0.001 |
| Neck complaint | Heat exposure | 1.05 (1.01–1.10) | 0.017 |
| Neck complaint | Cold exposure | 1.07 (1.02–1.13) | 0.010 |
| Shoulder complaint | Traffic exposure | 1.11 (1.06–1.16) | <0.001 |
| Shoulder complaint | Air pollution exposure | 1.03 (0.99–1.07) | 0.119 |
| Shoulder complaint | Noise exposure | 1.02 (0.99–1.06) | 0.185 |
| Shoulder complaint | Road irregularity/vibration exposure | 1.08 (1.04–1.13) | <0.001 |
| Shoulder complaint | Heat exposure | 1.03 (0.99–1.07) | 0.172 |
| Shoulder complaint | Cold exposure | 1.01 (0.96–1.07) | 0.676 |
| Low back complaint | Traffic exposure | 1.16 (1.12–1.20) | <0.001 |
| Low back complaint | Air pollution exposure | 1.07 (1.04–1.11) | <0.001 |
| Low back complaint | Noise exposure | 1.04 (1.01–1.07) | 0.004 |
| Low back complaint | Road irregularity/vibration exposure | 1.10 (1.07–1.13) | <0.001 |
| Low back complaint | Heat exposure | 1.05 (1.01–1.08) | 0.003 |
| Low back complaint | Cold exposure | 1.03 (1.00–1.07) | 0.086 |

Reduced-adjustment models included age, courier experience, work type, daily work duration, daily riding duration, break frequency, and package weight. Sleep duration, self-rated stress score, and physical activity ≥150 min/week were omitted. Estimates are presented as PRs with 95% CIs, expressed per 1-point increase on the 0–10 self-reported exposure scale. *p* values are shown for each estimate.

**Supplementary Table S6.** Tertile-based associations between self-reported working-condition indicators and 7-day complaints

| **Outcome** | **Exposure** | **Contrast** | **PR (95% CI)** | ***p*** |
| --- | --- | --- | --- | --- |
| Neck complaint | Traffic exposure | Medium vs Low | 1.76 (1.31–2.37) | <0.001 |
| Neck complaint | Traffic exposure | High vs Low | 1.75 (1.27–2.41) | <0.001 |
| Neck complaint | Air pollution exposure | Medium vs Low | 1.71 (1.28–2.28) | <0.001 |
| Neck complaint | Air pollution exposure | High vs Low | 1.64 (1.22–2.20) | 0.001 |
| Neck complaint | Noise exposure | Medium vs Low | 1.55 (1.21–1.99) | <0.001 |
| Neck complaint | Noise exposure | High vs Low | 1.40 (1.06–1.85) | 0.018 |
| Neck complaint | Road irregularity/vibration exposure | Medium vs Low | 1.64 (1.22–2.18) | <0.001 |
| Neck complaint | Road irregularity/vibration exposure | High vs Low | 1.55 (1.12–2.15) | 0.008 |
| Neck complaint | Heat exposure | Medium vs Low | 0.99 (0.77–1.28) | 0.961 |
| Neck complaint | Heat exposure | High vs Low | 1.35 (1.06–1.72) | 0.015 |
| Neck complaint | Cold exposure | Medium vs Low | 1.45 (1.09–1.93) | 0.011 |
| Neck complaint | Cold exposure | High vs Low | 1.51 (1.11–2.05) | 0.009 |
| Shoulder complaint | Traffic exposure | Medium vs Low | 1.53 (1.13–2.09) | 0.006 |
| Shoulder complaint | Traffic exposure | High vs Low | 1.76 (1.25–2.46) | 0.001 |
| Shoulder complaint | Air pollution exposure | Medium vs Low | 1.33 (1.02–1.72) | 0.034 |
| Shoulder complaint | Air pollution exposure | High vs Low | 1.29 (0.98–1.70) | 0.075 |
| Shoulder complaint | Noise exposure | Medium vs Low | 1.36 (1.06–1.73) | 0.014 |
| Shoulder complaint | Noise exposure | High vs Low | 1.06 (0.80–1.40) | 0.674 |
| Shoulder complaint | Road irregularity/vibration exposure | Medium vs Low | 1.61 (1.17–2.22) | 0.004 |
| Shoulder complaint | Road irregularity/vibration exposure | High vs Low | 1.78 (1.26–2.50) | 0.001 |
| Shoulder complaint | Heat exposure | Medium vs Low | 0.87 (0.67–1.13) | 0.307 |
| Shoulder complaint | Heat exposure | High vs Low | 1.20 (0.94–1.53) | 0.139 |
| Shoulder complaint | Cold exposure | Medium vs Low | 0.85 (0.64–1.13) | 0.261 |
| Shoulder complaint | Cold exposure | High vs Low | 1.17 (0.89–1.54) | 0.270 |
| Low back complaint | Traffic exposure | Medium vs Low | 2.00 (1.57–2.56) | <0.001 |
| Low back complaint | Traffic exposure | High vs Low | 2.16 (1.68–2.78) | <0.001 |
| Low back complaint | Air pollution exposure | Medium vs Low | 1.42 (1.16–1.72) | <0.001 |
| Low back complaint | Air pollution exposure | High vs Low | 1.44 (1.18–1.76) | <0.001 |
| Low back complaint | Noise exposure | Medium vs Low | 1.26 (1.04–1.51) | 0.016 |
| Low back complaint | Noise exposure | High vs Low | 1.20 (0.99–1.45) | 0.060 |
| Low back complaint | Road irregularity/vibration exposure | Medium vs Low | 1.81 (1.42–2.30) | <0.001 |
| Low back complaint | Road irregularity/vibration exposure | High vs Low | 1.83 (1.42–2.36) | <0.001 |
| Low back complaint | Heat exposure | Medium vs Low | 1.09 (0.90–1.31) | 0.381 |
| Low back complaint | Heat exposure | High vs Low | 1.25 (1.06–1.48) | 0.008 |
| Low back complaint | Cold exposure | Medium vs Low | 1.01 (0.82–1.23) | 0.942 |
| Low back complaint | Cold exposure | High vs Low | 1.17 (0.96–1.42) | 0.126 |

Each exposure indicator was categorized into tertiles, with the low tertile used as the reference group. Models were adjusted for age, courier experience, work type, daily work duration, daily riding duration, break frequency, package weight, sleep duration, self-rated stress score, and physical activity ≥150 min/week. Estimates are presented as prevalence ratios with 95% confidence intervals. Tertile cut-points were based on the distribution of each self-reported working-condition indicator in the analytic sample. CI, confidence interval; PR, prevalence ratio.

**Supplementary Table S7.** Trend analyses for tertile-based exposure models

| **Outcome** | **Exposure** | **PR per tertile increase (95% CI)** | ***p* for trend** | **Pattern assessment** |
| --- | --- | --- | --- | --- |
| Neck complaint | Traffic exposure | 1.31 (1.13–1.51) | <0.001 | Possible plateau or non-linear pattern |
| Neck complaint | Air pollution exposure | 1.28 (1.11–1.46) | <0.001 | Possible plateau or non-linear pattern |
| Neck complaint | Noise exposure | 1.18 (1.04–1.35) | 0.010 | Possible plateau or non-linear pattern |
| Neck complaint | Road irregularity/vibration exposure | 1.22 (1.06–1.41) | 0.006 | Possible plateau or non-linear pattern |
| Neck complaint | Heat exposure | 1.15 (1.01–1.31) | 0.033 | Possible threshold effect at higher exposure |
| Neck complaint | Cold exposure | 1.21 (1.05–1.40) | 0.009 | Monotonic increase / broadly linear pattern |
| Shoulder complaint | Traffic exposure | 1.31 (1.12–1.54) | <0.001 | Monotonic increase / broadly linear pattern |
| Shoulder complaint | Air pollution exposure | 1.14 (1.00–1.30) | 0.053 | Possible plateau or non-linear pattern |
| Shoulder complaint | Noise exposure | 1.04 (0.92–1.18) | 0.533 | Possible plateau or non-linear pattern |
| Shoulder complaint | Road irregularity/vibration exposure | 1.30 (1.12–1.52) | <0.001 | Monotonic increase / broadly linear pattern |
| Shoulder complaint | Heat exposure | 1.08 (0.95–1.23) | 0.248 | No clear monotonic pattern |
| Shoulder complaint | Cold exposure | 1.10 (0.95–1.27) | 0.208 | No clear monotonic pattern |
| Low back complaint | Traffic exposure | 1.45 (1.29–1.63) | <0.001 | Monotonic increase / broadly linear pattern |
| Low back complaint | Air pollution exposure | 1.21 (1.10–1.34) | <0.001 | Monotonic increase / broadly linear pattern |
| Low back complaint | Noise exposure | 1.10 (1.00–1.21) | 0.042 | Possible plateau or non-linear pattern |
| Low back complaint | Road irregularity/vibration exposure | 1.32 (1.19–1.47) | <0.001 | Monotonic increase / broadly linear pattern |
| Low back complaint | Heat exposure | 1.12 (1.02–1.22) | 0.016 | Monotonic increase / broadly linear pattern |
| Low back complaint | Cold exposure | 1.08 (0.98–1.19) | 0.118 | Numerically increasing pattern; trend not statistically significant |

Trend analyses were performed by entering exposure tertiles as ordinal variables in adjusted modified Poisson models. Estimates are presented as prevalence ratios with 95% confidence intervals. *p* for trend indicates the *p* value for ordinal trend across tertiles. Pattern assessment was based on comparison of the medium versus low and high versus low tertiles to evaluate whether the observed associations were broadly monotonic, suggestive of a threshold effect, or suggestive of a plateau/non-linear pattern. CI, confidence interval; PR, prevalence ratio.
